# The Importance of Interfaces in Multi‐Material Biofabricated Tissue Structures

**DOI:** 10.1002/adhm.202101021

**Published:** 2021-09-12

**Authors:** Martina Viola, Susanna Piluso, Jürgen Groll, Tina Vermonden, Jos Malda, Miguel Castilho

**Affiliations:** ^1^ Department of Orthopeadics University Medical Center Heidelberglaan 100 Utrecht 3508 GA The Netherlands; ^2^ Department of Pharmaceutics Utrecht Institute for Pharmaceutical Sciences (UIPS) Faculty of Science Utrecht University Utrecht 3508 TB The Netherlands; ^3^ Department of Functional Materials in Medicine and Dentistry at the Institute of Functional Materials and Biofabrication and Bavarian Polymer Institute University of Würzburg Pleicherwall 2 D‐97070 Wurzburg Germany; ^4^ Department of Clinical Sciences Faculty of Veterinary Medicine Utrecht University Yalelaan 1 Utrecht 3584 CL The Netherlands; ^5^ Department of Biomedical Engineering Eindhoven University of Technology De Zaale Eindhoven 5600 MB The Netherlands

**Keywords:** bioinks, composites, hybrid bioprinting, materials interfaces, multitechnology biofabrication, regenerative medicine, reinforcement

## Abstract

Biofabrication exploits additive manufacturing techniques for creating 3D structures with a precise geometry that aim to mimic a physiological cellular environment and to develop the growth of native tissues. The most recent approaches of 3D biofabrication integrate multiple technologies into a single biofabrication platform combining different materials within different length scales to achieve improved construct functionality. However, the importance of interfaces between the different material phases, has not been adequately explored. This is known to determine material's interaction and ultimately mechanical and biological performance of biofabricated parts. In this review, this gap is bridged by critically examining the interface between different material phases in (bio)fabricated structures, with a particular focus on how interfacial interactions can compromise or define the mechanical (and biological) properties of the engineered structures. It is believed that the importance of interfacial properties between the different constituents of a composite material, deserves particular attention in its role in modulating the final characteristics of 3D tissue‐like structures.

## Graphical abstract

Converged biofabrication allows the in vitro fabrication of biological constructs by precise combination of materials and cells. However, material's interactions, which are known to determine biofabricated constructs’ performance, have not been adequately investigated. This review examines the interface between different material phases in (bio)fabricated structures, with a particular focus on how interfacial interactions can define their mechanical and biological properties.

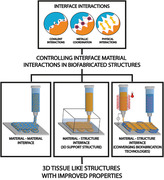

## 1 Introduction

Biofabrication is a rapidly evolving field that uses additive manufacturingtechniques to create biological constructs with precise control over the placement of cells and biomaterials.^[^ ^1^, ^2^ ^]^ The underlying hypothesis is that, by giving cells a defined bioinspired spatial organization, appropriate conditions are generated to facilitate cell‐cell and cell‐material interactions and subsequent biological maturation.^[^ ^3^, ^4^ ^]^ These constructs can find application in the delivery or in the screening of drugs and as implants to restore or replace damaged tissues.^[^ ^5^ ^]^ Despite great progress in the field, the generation of functional biofabricated structures and their effective application in vivo is at a standstill.^[^ ^6^ ^]^

In the last decade, the development of biopolymer‐based hydrogels for biofabrication has gained particular attention. Hydrogels are composed of networks of hydrophilic polymer chains that are able to absorb high quantities of water (up to 90% of their volume),^[^ ^7^ ^]^ are highly cyto‐compatible and therefore exhibit great similarities to the non‐fibrillar part of the natural extracellular matrix of living tissues. These materials must guarantee a cytocompatible environment that is not excessively stiff, which allows efficient diffusion of nutrients and removal of metabolic waste, but at the same time should be mechanically stable to provide structural support.^[^ ^1^, ^8^ ^]^ In the context of biofabrication, we should highlight the difference in definitions proposed by Groll et al. between biomaterial ink and bioink, the first one is just an aqueous formulation of hydrogel precursors that can become the second one by addition of cells.^[^ ^7^ ^]^ Despite the attractive characteristics of many hydrogel platforms, their poor mechanical properties limit their actual use, especially in tissues submitted to challenging mechanical loading environments, for example tissues in the musculoskeletal system.^[^ ^9^, ^10^ ^]^

Alternatively, structures with higher mechanical stability have been explored using stronger and tougher hydrogel materials^[^ ^11^, ^12^ ^]^ whose environment does not favor cell adhesion.^[^ ^13^ ^]^ The specific environmental needs of the embedded cells, which thrive best in dilute networks, are in contrast with the required mechanical properties for biofabrication processing and tissue repair, which improve with higher polymer concentration and higher crosslinking density.^[^ ^14^, ^15^ ^]^ Composites of soft hydrogel‐based materials reinforced with nanofillers, particles, fibers, or support structures are therefore considered a good strategy to improve the overall mechanical performance of biofabricated constructs.^[^ ^16^, ^17^, ^18^ ^]^ Here, the term composite refers to the combination of multiple materials with properties that are most often different from each other, but when combined can improve the final overall characteristics of the composite construct.^[^ ^19^ ^]^ Particular attention has recently being paid to the possibility of creating hydrogel‐based composites by using biofabrication technologies.^[^ ^8^, ^13^, ^20^, ^21^, ^22^ ^]^

To date, the majority of biofabrication technologies investigated to create composites are based on the use of single deposition methods, such as extrusion,^[^ ^23^ ^]^ inkjet,^[^ ^24^ ^]^ or laser‐assisted printing.^[^ ^25^ ^]^ Each of these methods has different working principles which are more suitable for certain classes of materials than others limiting material choice.^[^ ^26^, ^27^, ^28^ ^]^ Recent new approaches are emerging that converge multiple biofabrication and/or 3D printing technologies into a single biofabrication platform^[^ ^20^, ^29^ ^]^ with the goal of increasing the freedom of material combination and local control over composition and structure across different length scales.^[^ ^30^ ^]^ This processing approach assumes a crucial role in the biofabrication of composite materials that better resemble the complexity of biological materials. However, the importance of interfaces between the different composite material phases, in particular when combined by biofabrication strategies, has not been adequately explored. This is known to determine material's interaction and ultimately their mechanical and biological performance.

**Figure 1:**
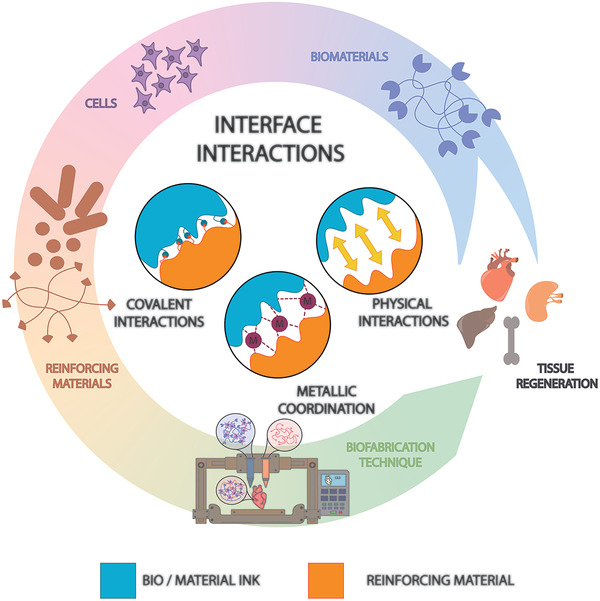
Schematic of principle material interface interactions and key players in biofabrication: from biomaterials up to the regeneration of tissues and organs.

In this review, we bridge this gap by critically examining the interface between different material phases in (bio)fabricated structures, with a particular focus on how interfacial interactions can compromise or define the mechanical (and biological) properties of the engineered structures. We start by reviewing the working principles of major material interface interactions, that is, metallic coordination, physical interactions, and covalent bonds, and then follow on discussing the most common methods of creating hydrogel composites and their respective interactions following a bottom up approach, that is, from material–material up to material–structure interfaces (**Figure** **1**). Important to note that we are aware that other important interactions exist, for example, between cells and materials, including its surface morphology, which can have a significant influence on cellular processes, including migration, proliferation, and tissue morphogenesis.^[^ ^31^, ^32^, ^33^, ^34^ ^]^ However, this is outside the scope of this review and we do refer the reader to other excellent works in the literature.^[^ ^35^, ^36^, ^37^, ^38^, ^39^, ^40^ ^]^ Taken together, we argue that understanding interfacial interactions between (reinforcing) materials and structures is essential to rationally design biofabricated biological constructs with improved mechanical and biological performance.

## 2 Interface Interactions

When engineering tissue constructs using different materials, combined or not by biofabrication techniques, interfaces between materials they are constituted of will occur. These interfaces will dictate the way that materials interact with each other and subsequently determine their mechanical performance and biological behavior. Despite the borderlines between material and structure are sometimes debatable, two types of interfaces can be considered if observed at different scales. Material‐material interfaces when observing at the nano‐ to micro‐scale, which refers to the interface between different material constituents of a composite; and material‐structure interfaces when observed at the micro‐ to macro‐scale, which refers to the interface between 3D printed structures and the reinforced material.^[^ ^41^ ^]^ Importantly, independent of the interface type, the conformational arrangement of the molecules at the interface regulates the chemical, physical and mechanical properties of composite materials, which is determined by the intermolecular forces that arise between the different materials.^[^ ^42^ ^]^ Such forces, like electrostatic forces, hydrophobic interactions, hydrogen bonds, coordination bonds, and covalent bonds, determine the orientation of the lateral functional groups of the molecules. Moreover, interactions can be weaker or stronger depending on their physico‐chemical nature, the stronger the interaction, the higher the mechanical energy required to detach two materials.^[^ ^42^ ^]^

Three types of materials interactions exist: chemical bonds, metallic, and physical interactions. In the formation of a chemical bond, the involved atoms switch from an unstable state with a high energy to a lower energy state, the amount of energy that is released is equivalent to the energy that will be needed to break the same bond.^[^ ^43^ ^]^ The chemical bonds are typically stronger and more stable than physical interactions. The metallic bond is slightly weaker than the chemical bond, it can occur thanks to the mutual attraction of opposite charges of two metals or between a metal and another charged atom, or thanks to the donation of an electron pair by the ligand. The physical bond, on the other hand, refers to the attractive forces that hold the molecules together, such as the Van der Waals forces or hydrogen bonds. This type of bond is the weakest and consequently the easiest to break, when compared to the chemical and metallic bonds.

In addition, besides the type of material interaction, molecular orientations at the materials interface are known to dictate the nature of the interactions between the components that make up a composite material.^[^ ^44^ ^]^ This feature is of considerable importance because chemical reactions that can occur at the interfaces, create a strong correlation between the chemical composition of the composite materials and their mechanical behavior.^[^ ^45^ ^]^ A detailed summary of the materials and manufacturing strategies to engineer material‐material and material‐structure interfaces is provided below.

## 3 Material–Material Interface

### 3.1 Nano‐Sized Filler Materials

A nanosized filler is defined as a material consisting of several phases, of which at least one is less than 100 nm in size.^[^ ^46^ ^]^ The substantial difference between composite materials with nanosized fillers and classic composite materials lies in the surface/volume ratio, which in the case of nanosized fillers is much higher. This results in a considerable effect on the interface interactions and consequent composite final properties, even for a relatively small amount of reinforcing material. In fact, the weight percentage of the reinforcement can be as low as 0.5–5%.^[^ ^47^ ^]^ The nanosized composite materials, in addition to their effect on the mechanical properties, can also improve their thermal stability and electrical conductivity properties.^[^ ^48^ ^]^ Typically, the geometry of nanosized fillers varies from platelets, to fibers or spheroids. Each nanofiller, except the spherical ones, is subject to anisotropy for which their characteristics change according to the assumed spatial orientation. In general, the following most common examples of nanofillers are inorganics,^[^ ^49^ ^]^ like metal‐based nanocomposites^[^ ^50^ ^]^ and ceramic‐based nanocomposites^[^ ^51^ ^]^ like graphene, graphene oxide,^[^ ^52^, ^53^ ^]^ carbon nanotubes, hydroxyapatite,^[^ ^54^ ^]^ silica nanoparticles,^[^ ^55^ ^]^ and polymer‐based nanocomposite materials.^[^ ^46^ ^]^

#### 3.1.1 Inorganic Nanosized Fillers

**Figure 2:**
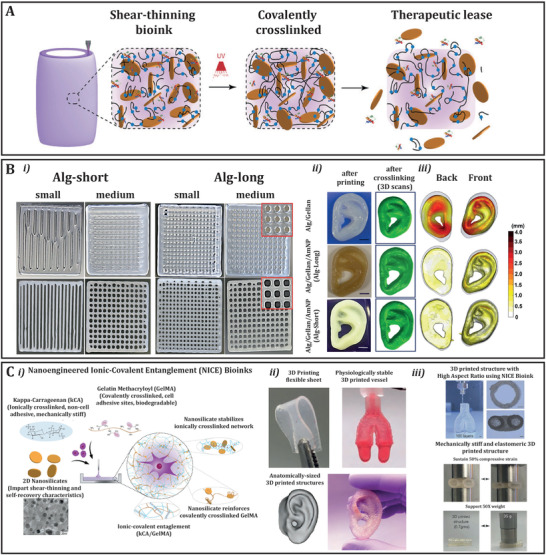
A) Controlled release of therapeutics by controlling interface of nanosilicates and poly(ethylene glycol)–dithiothreitol (PEGDTT) bioink in biofabricated 3D structures. The high surface area and charged characteristics of nanosilicates can sequester protein therapeutics within 3D printing structure. Reproduced with permission.^[^ ^60^ ^]^ Copyright 2019, Wiley‐VCH GmbH. B) Printability and printing fidelity of prepared inks; i) Left 4 images: Grid structures with small (1 layer) or medium (2 layers) spacing printed using Alg/gellan inks with/without AmNPs (with Alg Short); Right 4 images: Grid structures with small (2 layer) or medium (2 layers) spacing printed using Alg/gellan inks with/without AmNPs (with Alg‐Long); ii) Images of printed ears and 3D scans of printed ears after cross‐linking. Scale bar = 5 mm; iii) digitalized images of cross‐linked ears. The color indicates dimension difference between the printed, crosslinked construct and the original 3D digital model. The gray background is the outline of the 3D design used. Reproduced with permission.^[^ ^62^ ^]^ Copyright 2018, American Chemical Society. C) NICE bioinks combine nanocomposite reinforcement and ionic‐covalent entanglement reinforcement mechanisms to create a bioink that is tough, elastic, and highly printable; i) NICE bioinks use nanosilicates to reinforce an ionic‐covalent entanglement hydrogel made from GelMA and *κ*CA, creating a dual reinforced hydrogel network. These interactions allow the NICE bioink to behave as a solid at low shear stresses and improve shear thinning characteristics during bioprinting; ii) 3D printed structures from NICE bioink are mechanically (film) and physiological (bifurcated vessel) stable and allow fabrication of complex shape geometries as a human sized ear; iii) NICE bioinks print freestanding hydrogel structures with high aspect ratios and high print fidelity (scale bar = 1 mm). Crosslinked structures are stiff and elastomeric and can support more than 50‐times their own weight. Reproduced with permission.^[^ ^65^ ^]^ Copyright 2018, American Chemical Society.

Amongst the different inorganics used to form nanofillers, the most common are hydroxyapatite and silica‐based materials. The first one is similar to the inorganic component of bone tissue, while the second one has been widely used thanks to the ability of tuning its morphology and surface chemistry, allowing to be chemically modified.^[^ ^56^ ^]^ For example, Paşcu et al. reported a nanocomposite material based on polyhydroxybutyrate*‐co‐*(3‐hydroxyvalerate) (PHBV) with nanoparticles of hydroxyapatite, which was able to stimulate the proliferation of osteoblasts and improve formation of bone‐like matrix after 28 days of in vitro culture when compared with plain PHBV.^[^ ^57^ ^]^ Despite the promising biological outcome, the interface between polymer and hydroxyapatite nanofiller was not explicit engineered, which resulted in a decrease of the composite elastic modulus with the increase in hydroxyapatite concentration. Further, Fourier‐transform infrared spectroscopy (FTIR) analysis demonstrated that neither hydrogen bonds nor chemical bonds were present between the polar groups of PHBV and hydroxyapatite. This explains the lack of improvement in mechanical performance when compared to PHBV alone.^[^ ^57^ ^]^ Within the class of silica‐based materials, nanosilicates (NS), which are ultrathin anisotropic nanomaterials with a high surface area and a consequent high potential of being functionalized, have been major focus of interest.^[^ ^58^ ^]^ In one example of the application of these fillers, Byambaa et al. reported the biofabrication of bone‐like architectures using two different types of GelMA‐based hydrogels, one of which filled with silicate nanoplatelets.^[^ ^59^ ^]^ The inclusion of NS resulted in an improvement of the osteogenic potential of the GelMA hydrogel, with a degree of mineralization directly dependent on the quantity of added NS. In particular, the incorporation of NS was shown to promote the expression of osteocalcin (OCN), a marker for osteoblast differentiation, to improve calcium deposition, which indicates deposition of mineralized matrix, and interestingly, to promote the expression of a‐SMA, an index of vascular maturation.^[^ ^59^ ^]^ In addition, NS have also been successfully combined with other hydrogel precursors like poly(ethylene glycol) (PEG)–based poly(ethylene glycol)–dithiothreitol (PEG‐DTT)^[^ ^60^ ^]^ and kappa‐carrageenan,^[^ ^61^ ^]^ resulting in a significant improvement in the mechanical properties of the resulting composite (**Figure** **2**A). Another interesting strategy to increase both mechanical properties and print fidelity of extruded printed bioinks, was reported by Lee et al. which involved the incorporation of cationic modified silica nanoparticles (<100 nm) into an anionic polymer blend, composed of alginate and gellan gum.^[^ ^62^ ^]^ The electrostatic interactions between the nanoparticles and the polymers resulted in a significant increase in printability due to an increase in viscosity (1062%), when compared to the pristine hydrogels (Figure 2B).^[^ ^62^ ^]^ In general, the printability and mechanical properties of the printed constructs are directly proportional to the concentration of the NS fillers.^[^ ^63^ ^]^ However, in extrusion‐based printing processes, the presence of fillers may cause an increase in shear forces which can consequently compromise cell viability.^[^ ^63^ ^]^

Furthermore, to demonstrate the importance of the interactions at the interface between NS filler and hydrogel materials, Xia et al. introduced silica nanoparticles with butyl acrylate moieties (SiO_2_‐g‐PBA) in a poly(acrylamide*‐co‐*lauryl methacrylate) (P(AAm*‐co‐*LMA)) hydrogel.^[^ ^64^ ^]^ The addition of potassium persulphate and N,N,N′,N′‐tetramethylethylenediamine, with an increase in temperature (30 °C), induced chemical crosslinking between nanoparticles and hydrogel. Impressively, this chemical interaction resulted in an increase of strain at fracture of ≈500%.^[^ ^64^ ^]^ Another interesting study, reported the development of an extrudable bioink composed of a GelMA and k‐carrageenan hydrogel reinforced with 2D Laponite XLG NS (nSi). The material itself showed elastomeric characteristics, thanks to the dissipation of energy through reversible disruption of ionic network crosslinks between the two polymer networks; while the incorporation of nSi, was associated with an improvement of stiffness and cell adhesion, thanks to the electrostatic interaction between the particles and the polymer networks (Figure 2C).^[^ ^65^ ^]^ While the mechanical benefit of incorporating inorganic nanosized fillers into hydrogel polymers is clear, there is still a lack of in‐depth knowledge on the relationship between the structure of the nanofiller and their relationship with the final properties of the material. Moreover, these nanofillers are not suitable for all types of regenerative applications^[^ ^66^ ^]^ and could in the long run lead to material rejection caused by the immune system.^[^ ^59^, ^67^, ^68^ ^]^ Therefore, understanding the interactions between the immune system and biomaterials is a crucial aspect. It is known that the implantation of a biomaterial induces an inevitable immune response, the outcome of which defines whether this material will be integrated into the host tissue.^[^ ^69^ ^]^ This topic has been investigated by multiple research groups. For example, Blum et al. reported on an in vitro model to analyze the immune response to peptide fractions Arg‐Gly‐Asp (RGD) immobilized on PCL nanofibers.^[^ ^70^ ^]^ The immune response, in particular due to the action of neutrophils, cleaved the binding site of the RGDS which consequently decreased cell adhesion of cultured human MSC. To prevent this immune response, the authors proposed a temporary protective coating composed of hyaluronic acid and adipic acid dihydrazide. This coating was able to block the immediate attack of neutrophils and degraded after ≈5 days, which favored subsequent cell adhesion.^[^ ^70^ ^]^ However, the attempt to replicate the effect of the immune response in vitro may not be the best strategy to ensure long‐term in vivo functionality of implanted biomaterials. Alternative strategies to engineer biomaterials interfaces to modulate in situ tissue regeneration are currently subject of intense research.

#### 3.1.2 Polymeric Nanocomposites

Polymeric nanocomposites (PNC) are probably the most used nanofillers in regenerative medicine. There are two main factors to consider when designing PNC, the physical compatibility of the materials and their degradation rate. Low compatibility between components can result in inhomogeneity of properties and phase separation, while unsynchronized degradation can lead to a premature collapse of the structure. To overcome the problem of incompatibility between the phases, without compromising the mechanical performance of the composite, Kai et al. developed a reinforced gelatin hydrogel composed of gelatin with nanofibers composed of a blend of gelatin and polycaprolactone (PCL).^[^ ^71^ ^]^ The nanofibers were prepared by a coaxial electrospinning process. Once cut and dispersed in the gelatin solution, the nanofibers were crosslinked with glutaraldehyde. What was interesting in this material is the improved resistance to degradation and the ability to approximate to the native ECM structure. In fact, this study not only demonstrated that the presence of microfibers does not compromise cell proliferation but facilitates it, providing binding sites for cell adhesion.^[^ ^71^ ^]^

Furthermore, to avoid the problem of compatibility between main polymer and reinforced phase, Rajkhowa et al. introduced a nanocomposite material using the same polymer, silk fibroin (SF), in the form of solution and particles prepared by salt leaching method,^[^ ^72^ ^]^ while Zhang et al. introduced quaternized tunicate cellulose nanocrystals (Q‐TCNCs) into a poly(acrylic acid*‐co‐*acrylamide) hydrogel (PAAAM).^[^ ^73^ ^]^ The Q‐TCNCs acted both as a reinforcement and as a crosslinker agent, where the positive charges interacted with the carboxyl groups (COO^−^) of a PAAAM). In the same study the authors demonstrated that the use of Fe^3+^ allows to form a coordination network with the COO^−^ groups of the PAAAM hydrogel. The introduction of Fe^3+^ resulted in a significant improvement on the nanocomposite material toughness when compared to Q‐TCNCs alone.^[^ ^73^ ^]^ Along the same vein, Shao et al. explored the use of Fe^3+^ ions to create a coordination network with cellulose nanofibrils (NFCs) and with poly(acrylic acid) (PAA).^[^ ^74^ ^]^ Both polymers were involved in the formation of coordination bonds with Fe^3+^, which resulted in a composite material with extraordinary elastic and self‐healing properties.^[^ ^74^ ^]^

Another interesting approach to improve the interface properties between the two polymers involves the use of surface treatments by plasma technologies. Li et al. designed a nanocomposite material composed of an hyaluronic acid (HA) hydrogel reinforced with PCL nanofibers.^[^ ^68^ ^]^ The PCL was treated with plasma to expose the carboxyl groups on the surface of PCL, which were then converted in thiol‐reactive maleimide groups (MAL‐PCL). These fibers were then dispersed in a thiolated hydrogel matrix (HA‐SH), which resulted in a significant reinforcement effect after crosslinking between the two polymer structures.

Despite great advances in the synthesis of nanocomposites and their combination with hydrogels, cyto‐ and bio‐compatibility are important concerns.^[^ ^75^ ^]^ Numerous studies have been carried out to understand the in vitro and in vivo behavior of these nanocomposites, although a complete understanding of their performance, as well as the effect of the interface properties, is still lacking.

### 3.2 Particles and Microfibers

**Figure 3:**
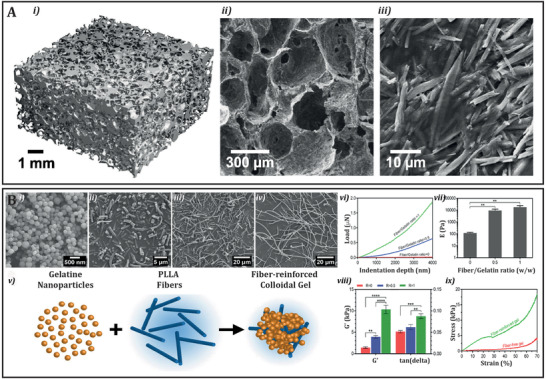
A) The hierarchical structure of Hydroxyapatite–collagen scaffolds reinforced with 80 vol% hydroxyapatite microparticles; i) micro‐CT reconstruction of collagen composite; ii) SEM micrograph of the internal microstructure showing interconnected pores, and iii) higher magnification SEM micrograph of the scaffold surface showing hydroxyapatite reinforcement. Reproduced with permission.^[^ ^81^ ^]^ Copyright 2015, Elsevier. B) Building blocks and formation of colloidal gels. Scanning electron micrographs of i) gelatin nanoparticles, and PLLA fibers aminolyzed for ii) 6 h, iii) 4 h, and iv) 3 h. Schematic illustration of the formation of fiber‐reinforced gels is shown in (v); vi) Load–displacement curves and vii) elastic modulus (E) of hydrogels with different fiber contents, measured by nanoindentation tests. ** indicates statistical difference (*p* < 0.005). The fiber‐reinforced gels were prepared using 4 h aminolyzed fibers; viii) Viscoelastic properties of the hydrogels after 7 days of cell culture. R indicates fiber/gelatin ratio (w/w). *, **, ***, and **** indicate statistical difference with *p* < 0.05, *p* < 0.01, *p* < 0.001, and *p* < 0.0001, respectively. The fiber‐reinforced gels were prepared using 4 h aminolyzed fibers; ix) Stress–strain curves of fiber‐reinforced and pure gelatin colloidal gels during compressive loading. The fiber‐reinforced gels were prepared at a fiber/gelatin (w/w) ratio of 1, using 4 h aminolyzed fibers. Reproduced with permission.^[^ ^83^ ^]^ Copyright 2018, Elsevier.

The incorporation of particles^[^ ^76^ ^]^ or microfibers^[^ ^77^, ^78^, ^79^, ^80^ ^]^ within hydrogels can have similar results as nano‐size fillers. However, unlike the latter, these are larger reinforcing structures that typically do not chemically interact with the hydrogel phase as demonstrated by different research groups. For example, Kane et al. combined micrometer sized hydroxyapatite particles with collagen fibers (**Figure** **3**A).^[^ ^81^ ^]^ The composite scaffold prepared by compression molding, showed an increase in compressive modulus of approximately one order of magnitude higher than the collagen‐only scaffold.^[^ ^81^ ^]^ Although no chemical interaction between the particles and hydrogel matrix was confirmed, the presence of the hydroxyapatite particles facilitated shapeability and shape retention of the collagen gels and importantly, significantly improved in vitro cell infiltration and differentiation on scaffolds with thicknesses up to a 3 mm.^[^ ^81^ ^]^

In a different approach, Kosik‐Kozioł et al., reported the combination of sub‐micrometer sized polylactide (PLA) fibers in an alginate‐based hydrogel bioink.^[^ ^82^ ^]^ These fibers had an approximate length of 67 ± 49 µm and were randomly distributed within the alginate ink without any preferential orientation. The rheological behavior of the resulting composite bioink indicated that PLA fibers could not flow at high shear rates, but remained associated with each other, probably through hydrophobic interactions.^[^ ^82^ ^]^ In a different study with a similar alginate hydrogel reinforced with PLA microfibers, an increase in stiffness of approximately four times (for a reinforcing microfibers volume fraction of 1–2%) was observed when compared with the alginate alone.^[^ ^82^ ^]^ Furthermore, Diba et al. reported on the reinforcement of a colloidal gel based on gelatin nanoparticles with poly‐L‐lactic acid (PLLA) micrometer size fibers of different lengths (Figure 3B).^[^ ^83^ ^]^ The incorporation of these microfibers resulted in an almost tenfold increase in the gel elastic modulus, from 2.4 ± 0.2 to 23.1 ± 2.1 kPa, when compared to the colloidal gelatin gel alone. This strategy of reinforcement allowed to obtain an injectable and mechanically strong colloidal gel.^[^ ^83^ ^]^

Further, using PLA as reinforcing material, Joshi et al. reported on the fabrication of hybrid scaffolds by combining electrospinning and casting processes.^[^ ^84^ ^]^ Fibers were obtained by electrospinning of *β*‐tricalcium phosphate‐incorporated‐PLA nanofibers and then dispersed in a GelMA hydrogel. The fiber reinforcement resulted in an increase in the mechanical performance of the composite constructs, from 7.5 to 45.37 kPa in compressive strength, as well as an enhancement of MSCs‐proliferation, and differentiation toward bone lineage.^[^ ^84^ ^]^

**Figure 4:**
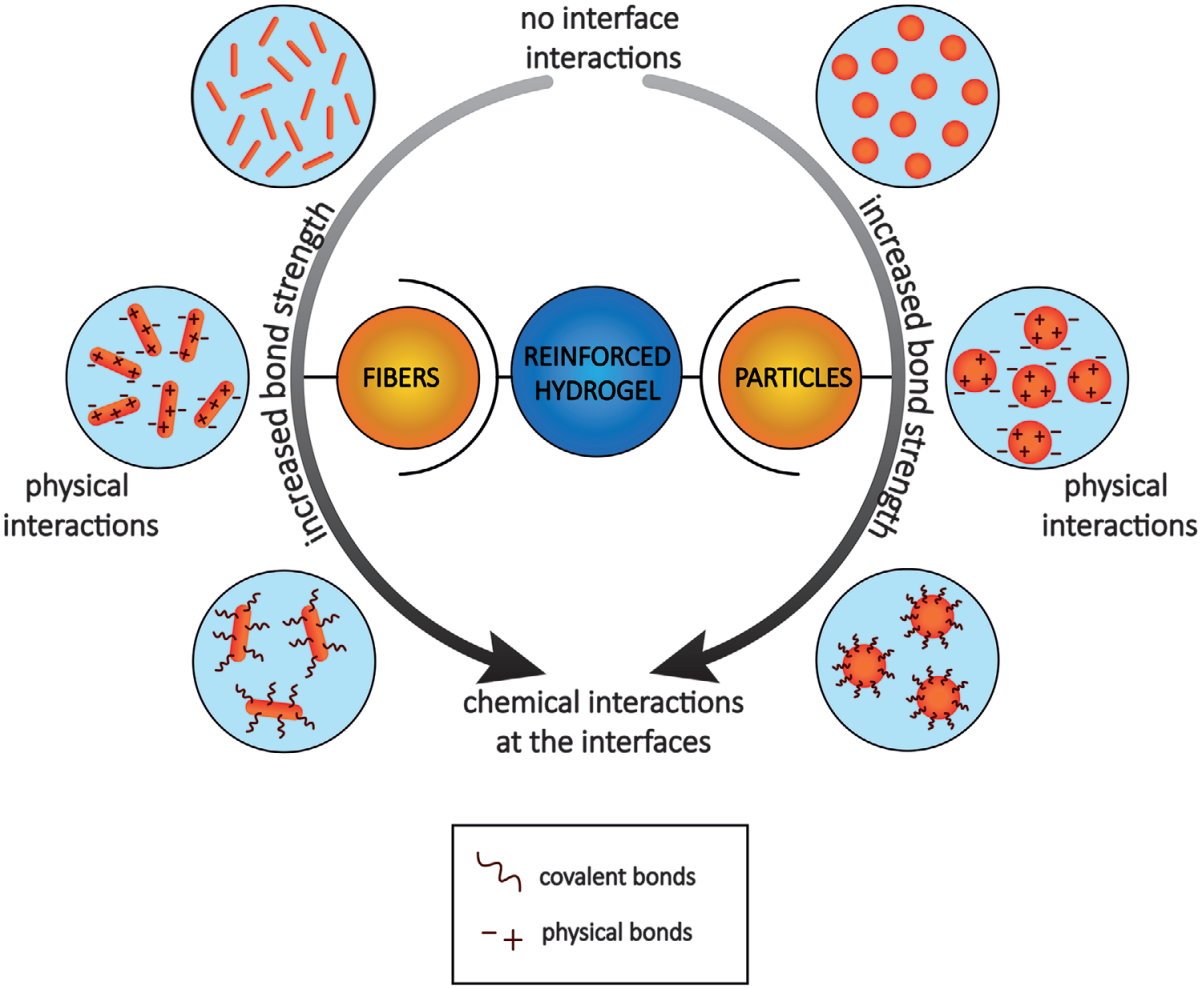
Schematic representation of most common interactions between nanofillers and hydrogels. Top: nanofillers without any interfacial interaction with the hydrogel‐based matrix. Middle: the physical interactions, such as hydrogen bonds. Bottom, covalent chemical bonds. From top to bottom an increase in mechanical stability is observed.

Thus, it seems clear that from nanofillers to particles and microfibers all have proven success in the reinforcement of hydrogels. However, the final properties of the composite material are highly dependent on the chemical nature of the material interactions as well as on the structural arrangement and distribution of the reinforcing material. In **Figure** **4**, we summarize the most common interactions that have been reported between nanofiller, particles and microfibers reinforcements, and hydrogels.

### 3.3 Chemical Reinforcement

Depending on the type of crosslinking, hydrogels can be distinguished in physical and chemical. Physical crosslinking is triggered by interactions such as ionic, hydrogen bonds, hydrophobic, and protein interaction. While chemical crosslinking can be achieved in the presence of functional lateral groups, thanks to chemical reactions of complementary groups, or reactions such addition, condensation, and high energy irradiation or enzymes, resulting in covalent bonds.^[^ ^85^ ^]^ Both crosslinking strategies have been explored to control the interaction between different hydrogel phases and with that modulate their biological and mechanical performance. This resulted in significant advancements along two main areas, interpenetrating polymer networks (IPNs) and double network hydrogels (DN) as summarized below.

#### 3.3.1 IPNs

**Figure 5:**
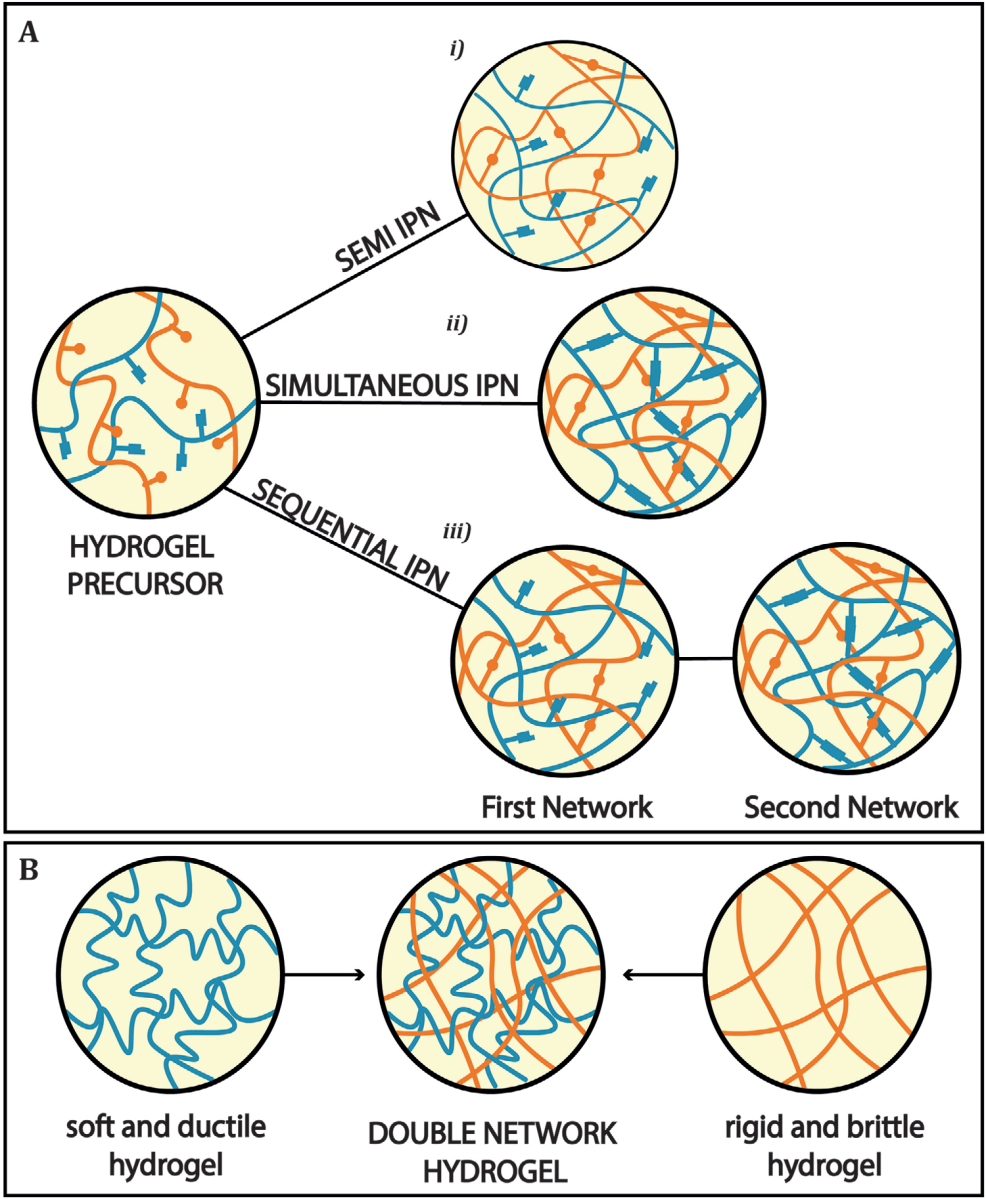
A) Schematic representation of interpenetrating polymer networks (IPNs) and double network hydrogels (DN); i) semi‐IPN, obtained by a mixture of polymers in which only one is cross‐linked and the other is trapped in the first network; ii) simultaneous IPN, where the precursors of the network are mixed together, and the network is formed at the same time; iii) sequential IPN in which where networks are formed one after the other. B) DN hydrogel, in which the two polymers have completely different properties.

An IPN can be described as two or more polymer networks intertwined in which, at least one, is synthesized, or crosslinked in the presence of the other.^[^ ^86^ ^]^ IPN hydrogels can be classified in semi‐IPN, simultaneous IPN, and sequential IPN. A semi‐IPN is made by a mixture of polymers in which only one is cross‐linked and the other is simply trapped in the first network (**Figure** **5**A‐i).^[^ ^87^ ^]^ In a simultaneous IPN, the precursors of the networks are mixed together and the networks are formed at the same time, without interfering with each other (Figure 5A‐ii). While in sequential IPN, the second network is formed after the first one (Figure 5A‐iii).^[^ ^88^ ^]^

IPNs are often created with the purpose of exploiting the properties of one of the components while preserving the characteristics of the other. In extraordinary cases, the IPN may show entirely new properties that were not observed in either of the two individual networks.^[^ ^87^ ^]^ For example, Suo et al. showed that by combining an IPN GelMA and chitosan, two widely used polymers, a significant increase in compression properties could be obtained when compared with each polymer phase individually.^[^ ^89^ ^]^ Such successful combination, can only be obtained when mutual miscibility of the components is considered. In general, when polymers do not mix well with each other, this could lead to phase separation of the resulting mixture. However, the crosslinking of at least one of the two components in the presence of the other provides a way to impose mixing of the two polymers.^[^ ^90^ ^]^ This provides nearly infinite combinations of polymers worth exploring as IPN for a variety of TE applications.^[^ ^91^ ^]^

#### 3.3.2 Double Network Hydrogels

DN can be seen as a subclass of IPNs that consist of two contrasting polymer networks, in which the first one is rigid and brittle, while the second is soft and ductile (Figure 5B).^[^ ^92^ ^]^ These contrasting properties between the two networks differentiate DN from IPNs and are responsible for strong double‐network hydrogels as described by different groups^[^ ^92^ ^]^ For example, Yang et al. reported on extrusion printing of a hydrogel precursor solution with shear thinning properties, poly(2‐acrylamido‐2‐methylpropanesulfonate).^[^ ^93^ ^]^ After printing, the scaffold was exposed to UV light to form a first hydrogel network. To create the second network, the produced scaffolds were soaked in a solution of polyacrylamide and subsequently UV cured. This strategy resulted in an increase of twofold in compression, tensile strength, and elastic modulus of the DN when compared to the single network gels individually. Notably, the authors observed that further tuning the ratio of the crosslinker in the first network and the amount of acrylamide in the second network, mechanical properties similar to those of articular cartilage could be obtained.^[^ ^93^ ^]^

Other materials including k‐carrageenan, polyacrylamide (PAAm), alginate, and PNIPAAm‐PEG have been explored to create DN hydrogels. Liu et al. exploited the physical gelation of k‐carrageenan to form the first network and used UV light to crosslink AAm and form the second network.^[^ ^94^ ^]^ This system was printable using an extrusion based method and showed promising mechanical properties with self‐healing characteristics.^[^ ^94^ ^]^ Moreover, Liang et al. reported a DN hydrogel composed of alginate and PNIPAAm‐PEG. The hydrogel mixture was extruded printed in a warm solution of Ca^2+^ to induce the gelation of the pre‐gel sample, triggered by the presence of calcium and temperature change.^[^ ^95^ ^]^ Although, FTIR showed no covalent bonding between the alginate and PNIPAAm‐PEG, the combination of these two materials allowed to overcome the brittleness of the alginate hydrogel and the low mechanical strength of PNIPAAm‐PEG. In addition, the DN alginate and PNIPAAm‐PEG hydrogel allowed encapsulation and maintenance of viable stem cells and an increase in cytocompatibility when compared to alginate alone, probably due to the high lipophilic environment of PNIPAAm‐PEG and the porous gel structure.^[^ ^95^ ^]^

Creation of dynamic covalent bonds is another area that attracts significant interest in the DN hydrogels. These types of covalent bonds are not only stronger than physical bonds but also provide reversible behavior. For example, Wang et al. designed a bioink based on hyaluronic acid modified with either hydrazine or aldehyde groups, that when mixed formed a covalent network with dynamic hydrazone bonds.^[^ ^96^ ^]^ These hyaluronic acid‐based hydrogels displayed shear thinning properties that are beneficial for extrusion 3D printing. A second network was composed of hyaluronic acid, but this time it was modified with norbornenes, which react with thiols in response to UV light. The second crosslinking strategy was responsible for the increase in stiffness of the printed scaffold.^[^ ^96^ ^]^ On a similar vein, Zhang et al. proposed a DN hydrogel composed of a chitosan (CS) network and poly(sulfobetaine*‐co‐*acrylic acid) (p(SBMA*‐co‐*AAc)) copolymer network.^[^ ^97^ ^]^ The first network was formed thanks to electrostatic interactions between CS and citrate ions, while of p(SBMA*‐co‐*AAc) network was formed by ionic interactions between zwitterionic moieties and hydrogen bonding between carboxyl groups. This DN hydrogel, besides having a higher resistance under deformation, high transparency, self‐healing properties, and good electrical conductivity, also showed shear thinning properties, demonstrating to be a promising material system for 3D printing applications.^[^ ^97^ ^]^

Clearly, DNs and IPNs can create dynamic networks, which invoke self‐assembly in living systems, thanks to non‐covalent interactions, but thanks also to covalent interactions they possess better mechanical properties than classic hydrogels.^[^ ^98^ ^]^ Although their cytocompatibility remains their most attractive feature for TE applications, their final mechanical properties still fall short of the ones observed in many (human) living tissues. The mechanical functionality of living tissues is often based on the presence of rigid fibrils organized in hierarchical structures.^[^ ^99^ ^]^ Such structures cannot be reproduced by any of these systems above‐mentioned alone. This has motivated the field to explore alternative reinforcement structures like 3D support structures with micro and macro sizes.^[^ ^100^ ^]^

## 4 Material–Structure Interface

It is known that the structural characteristics of the reinforcing material, predominately its volume fraction, porosity, and pore interconnectivity, determine the composite material's final properties. For example, reinforcing structures with high‐volume fractions typically result in enhancement of composite strength, while low volume fractions result in improved compliance.^[^ ^101^ ^]^ For example, Tonsomboon et al.,^[^ ^79^ ^]^ showed that the presence of reinforcing fibers could help improving composite fracture toughness; while Formica et al. demonstrated that organization and porosity of reinforcing structure are fundamental to improve composite strength and stiffness.^[^ ^102^ ^]^ Moreover, high‐volume fractions of reinforcing material typically result in stress shield effects which consequently compromise cell differentiation and neo‐tissue deposition, while low volume fractions typically favor neo‐tissue deposition due to facilitated mechano‐regulation aspects. In addition, high‐volume fractions of reinforcing material, which subsequently result in increased interfacial area between reinforcing structure and cell‐laden material, are essential to facilitate cell attachment points and potential guide new tissue growth.^[^ ^103^, ^104^ ^]^ Below, we discuss key material–reinforcing structure interfaces, their role in improving mechanical properties of composite constructs, as well as on supporting better regenerative outcomes.

### 4.1 3D Support Structures

An interesting approach to improve the mechanical properties of soft hydrogels involves the use of 3D reinforcing structures, able to address mechanical demanding environments, without interfering with the capacity to provide suitable conditions for cell survival and neo tissue formation.^[^ ^105^ ^]^ The most popular reinforcing structures include 3D lattices, immersed within a hydrogel, typically composed of thermoplastic polymers. For example, Xianang et al. reported the use of a knitted poly(L‐lactide*‐co‐*glycolide) (PLGA) mesh to reinforce a collagen‐chitosan hydrogel (CCS).^[^ ^106^ ^]^ Interestingly, the incorporation of PLGA into CCS hydrogel not only acted as a mechanical reinforcement but also facilitated cell adhesion, due to an increase in surface area, and induced the formation of blood vessels, due to porous structure created by the PLGA within the CCS gel. These findings were observed by implanting the PLGA reinforced CCS hydrogel scaffold in Sprague–Dawley rats for 4 weeks.

Furthermore, to enhance the compressive properties of soft cell laden hydrogels, Visser et al. introduced a novel strategy based on the use of well‐organized reinforcing polymeric fibers produced by melt‐electrowriting (MEW).^[^ ^107^ ^]^ Following a similar approach, de Ruijter et al. showed how the inclusion of melt electrowritten PCL sinusoidal reinforcing fibers within a poly(2‐hydroxyethyl methacrylate) (pHEMA) hydrogel resulted in the increase of the shear properties.^[^ ^108^ ^]^ Other authors have also contributed milestone works on the mechanical benefits of combining well organized microfiber scaffolds with soft (cell‐laden) hydrogels.^[^ ^109^, ^110^, ^111^ ^]^ For example, Castilho et al. proposed a 3D structure based on cell‐laden GelMA hydrogel reinforced with melt electrowritten bi‐layered PCL fiber scaffold, which approximated to the mechanical functionality of the superficial tangential zone and middle and deep zone (MDZ) of the native cartilage.^[^ ^112^ ^]^ The authors, also showed that the well‐integrated reinforcement structure within the chondrocyte laden gelMA hydrogel was able to promote new cartilaginous tissue formation upon in vitro physiologically relevant mechanical stimulation without the need for growth factors supplementation. On a different study, Schipani et al. combined alginate‐GelMA IPNs with PCL fibers printed by fused deposition modeling (FDM) to mimic the collagen structure found in native articular cartilage tissue.^[^ ^113^ ^]^ The reinforced hydrogel showed mechanical properties in the range of articular cartilage and supported chondrogenesis after 6 weeks in vitro culture^[^ ^113^ ^]^ Likewise, Mancini et al. showed that a PCL reinforced thiol‐ene crosslinkable hyaluronic acid/poly(glycidol)hydrogel (HA‐SH/P(AGE*‐co‐*G)) hybrid hydrogel loaded with stromal mesenchymal‐type cells (MSCs) and articular cartilage progenitor cells (ACPCs) was able to survive in vivo loading after 6 months implantation in an equine model.^[^ ^114^ ^]^

Despite the impressive contributions made by the incorporation of 3D printed reinforcement structures in soft hydrogels, this approach presents intrinsic limitations. For example, reinforcing structures obtained by extrusion printing methods typically involve the use of a high polymer volume fractions that may shield cells from beneficial mechanical loads. Additionally, the limited resolution of the standard extrusion methods has also been shown to limit control over local structural and consequent mechanical properties of reinforced constructs. On the other side, reinforcing scaffolds obtained by techniques that combine extrusion printing with electrical fields, opened new perspectives to solve these limitations but still lack an adequate interaction between the reinforcement structure and the hydrogel. This absence of an engineered interface typically results in heterogeneous material combination and anisotropic mechanical properties, uncontrolled degradation behavior and importantly limited cell adhesion points and new tissue deposition.

#### 4.1.1 Support Structure–Hydrogel Interface

**Figure 6:**
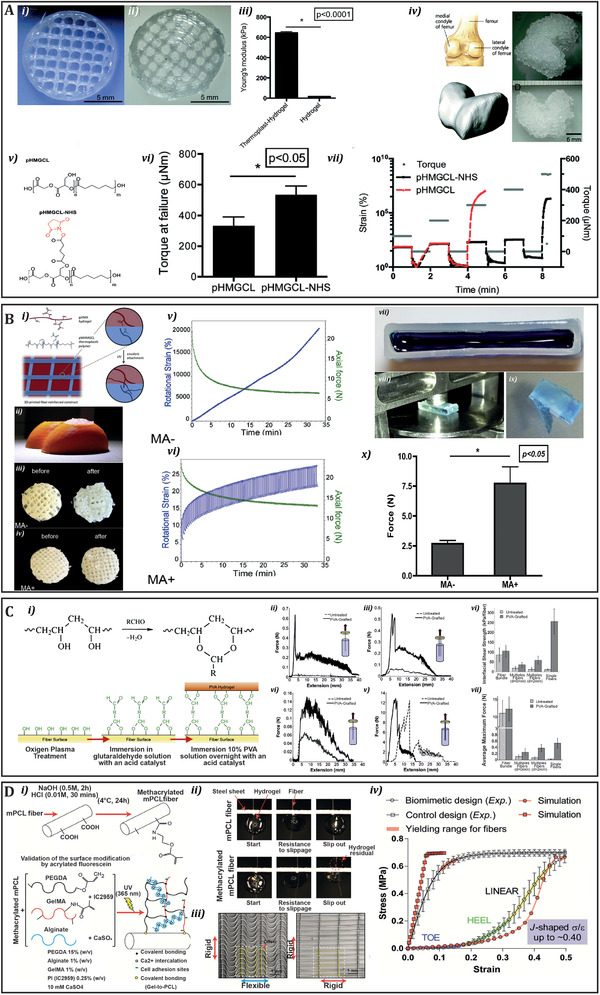
A) i) pHMGCL–NHS 3D‐printed network; ii) pHMGCL–NHS–PNC–PEGNHS thermoplast–hydrogel construct; iii) Young's modulus measured of hydrogel alone and reinforced constructs; iv) Schematic display of femoral condyles. Adapted from the American Heritage Dictionary. CAD model of femoral condyle and 3D printing of PNC–PEG NHS hydrogels in a condyle shape; v) Chemical structure of pHMGCL and pHMGCL‐NHS; vi) Evaluation of hydrogel–thermoplastic integration under creep‐recovery tests; vii) Torque at failure for PEG–pHMGCL and PEG–pHMGCL–NHS constructs (*n* = 3). Reproduced with permission.^[^ ^115^ ^]^ Copyright 2015, Royal Society of Chemistry. B) i) Chemical structure at the interface; ii) 3D printed model of a human knee femoral condyles (orange), where fiber reinforced constructs were press‐fit; iii) MA‐ and iv) MA+ scaffolds before and after the applied forces; Effect of 100 cycles of rotational forces on the rotational strain and axial force of v) MA‐ and vi) MA+ scaffolds; vii) Hollow cuboids printed from MA‐ and MA+ and filled with gelMA (stained blue); viii) compression was performed until ix) gelMA being squeezed out of the construct; x) respective force at construct failure. Reproduced with permission.^[^ ^116^ ^]^ Copyright 2014, Elsevier. C) i) Chemical cross‐linking of PVA; ii) Representative force vs. extension curves for both untreated and PVA‐grafted fibers for fiber pull‐out samples as a function of inter‐fiber spacing single fiber, iii) three fibers spaced 0 mm apart and iv) 2 mm apart in a triangular format, v) 60 fibers without any spacing; vi) Interfacial shear strength normalized to the number of fibers per sample and vii) average maximum force as a function of fiber formation. Reproduced with permission.^[^ ^117^ ^]^ Copyright 2014, Elsevier. D) i) Schematic representation of the surface methacrylation of MEW PCL fibers and composition of the IPN system; ii) Images of pull out tests comparing the adhesion of hydrogel droplets onto methacrylated and untreated PCL fibers; iii) FE‐SEM images of fibrous networks with biomimetic and control linear designs; iv) Experimental and numerical predictions of soft network composites under uniaxial tensile tests. Reproduced with permission.^[^ ^118^ ^]^ Copyright 2017, American Chemical Society.

As discussed in the previous section, the incorporation of reinforcement structures can increase the mechanical performance of soft hydrogels. However, the interactions at the interface between the reinforcing support structures and the hydrogel are quite often neglected. To address this, Boere et al. proposed a strategy to improve the mechanical integration between hydrogel and reinforcing material based on the conjugation of a hydrogel with poly(hydroxy‐methylglycolide*‐co‐ε*‐caprolactone) (pHMGCL) fibers functionalized with *N*‐hydroxysuccinimide (NHS) groups.^[^ ^115^ ^]^ A nearly 600‐fold increase in the Young's modulus of the reinforced hydrogel construct was obtained when compared to the hydrogel alone. Likewise, recovery experiments showed that the functionalization of the pHMGCL‐NHS improved the integration between the reinforcement and the hydrogel, likely due to the chemical crosslinking between the NHS groups and the free cysteines on the hydrogel (**Figure** **6**A).^[^ ^115^ ^]^ Later one, to further improve the integration between reinforcing material and hydrogel, poly(hydroxymethylglycolide*‐co‐ε*‐caprolactone) was functionalized with methacrylate groups and then blended with PCL, followed by GelMA hydrogel casting and formation through photo‐crosslinking. After applying a rotational force, in a model resembling an articular cartilage defect, the crosslinked material presented significantly higher mechanical resistance compared to the non‐crosslinked one (Figure 6B). Interestingly, after 6 weeks of in vitro culture, the composite construct supported the deposition of GAGs and type II collagen of seeded chondrocytes.^[^ ^116^ ^]^ Along a similar vein, Bas et al. developed a PCL‐fiber reinforced poly(ethylene glycol)/heparin (sPEG/Hep) hydrogel, in which PEG was thiol‐end modified and heparin was functionalized with maleimide moieties, to ensure cross linking through Michael addition.^[^ ^111^ ^]^ Impressively, the mechanical properties of the PCL reinforced sPEG/Hep hydrogel reached compression properties close to native cartilage.^[^ ^111^ ^]^ Following a different strategy, Holloway et al. studied the interfacial properties of an ultra‐high‐molecular‐weight polyethylene (UHMWPE) fiber‐reinforced poly(vinyl alcohol) (PVA) hydrogel.^[^ ^117^ ^]^ UHMWPE fibers have an inert and hydrophobic nature, characterized by poor cell adhesion. To improve the interfacial properties between UHMWPE fibers and PVA hydrogel, PVA was functionalized with aldehyde groups and grafted to plasma treated UHMWPE fibers. After grafting the interfacial shear strength increased from 11.5 ± 2.9 to 256.4 ± 64.3 kPa (Figure 6C).^[^ ^117^ ^]^ In another interesting work, Bas et al. modified the surface of a PCL‐based reinforcing structure by grafting photo‐crosslinkable acryl groups, which allowed the crosslinking between these groups and the IPN poly(ethylene glycol) diacrylate (PEGDA)/Alginate matrix (Figure 6D‐i). The authors showed that the interfacial shear strength of the crosslinked scaffold was three times higher than the non‐crosslinked one (untreated PCL) (Figure 6D‐ii), and that the composite material, with bioinspired reinforcing fibers, resembled collagen fibrils in biological materials (Figure 6D‐iii,iv).^[^ ^118^ ^]^ These results highlight well the importance of engineering the interfacial interactions between the constituent materials of a composite to improve both its mechanical and biological performance. In general, we observe that covalent grafting between the thermoplastic and the hydrogel‐based material is the preferred option, as it provides the more stable interface.

#### 4.1.2 Support Structure–Cells Interface

Material properties, such as stiffness, elasticity, and hydrophobicity can influence certain cellular behaviors.^[^ ^119^ ^]^ As mentioned in previous Section 4.1, the hydrophobicity of most of thermoplastic polymers processed with FDM or MEW 3D printing technologies, results in poor interactions with cells and other biological components. To overcome this, Bertlein et al. reported a strategy to permanently hydrophilize PCL with a NCO‐poly(ethylene oxide‐*stat*‐propylene oxide) (sP(EO‐*stat*‐PO)) coating. This strategy improved cell interaction and was able to form covalent bonds with bioactive molecules through the reaction with isocyanates.^[^ ^120^ ^]^ Following a similar approach, Blum et al. coated the PCL fiber scaffold with an ECM suspension composed of decellularized adipose tissue (DAP), fibronectin (FN), or laminin (LN).^[^ ^121^ ^]^ This coating was applied without the use of any chemical cross‐linker which might have potentially altered the biological properties of the ECM. Interestingly, the authors showed that the presence of DAP coating induced a more pronounced adipogenesis of hMSCs while compared to FN and LN coating only.^[^ ^121^ ^]^ Along the same vein, Bertlein et al. described a method for coating PCL melt electrowritten scaffolds with extracellular matrix derived components, that is, FN and gelatin (G), to guide the formation of capillary vascular structures. The authors showed that thicker neo‐vascular structures were obtained on FN coated PCL scaffold seeded with endothelial cells, while compared to endothelial cell sheets without coated support scaffold.^[^ ^122^ ^]^

### 4.2 Role of Converging (BIO) Fabrication Technologies

**Figure 7:**
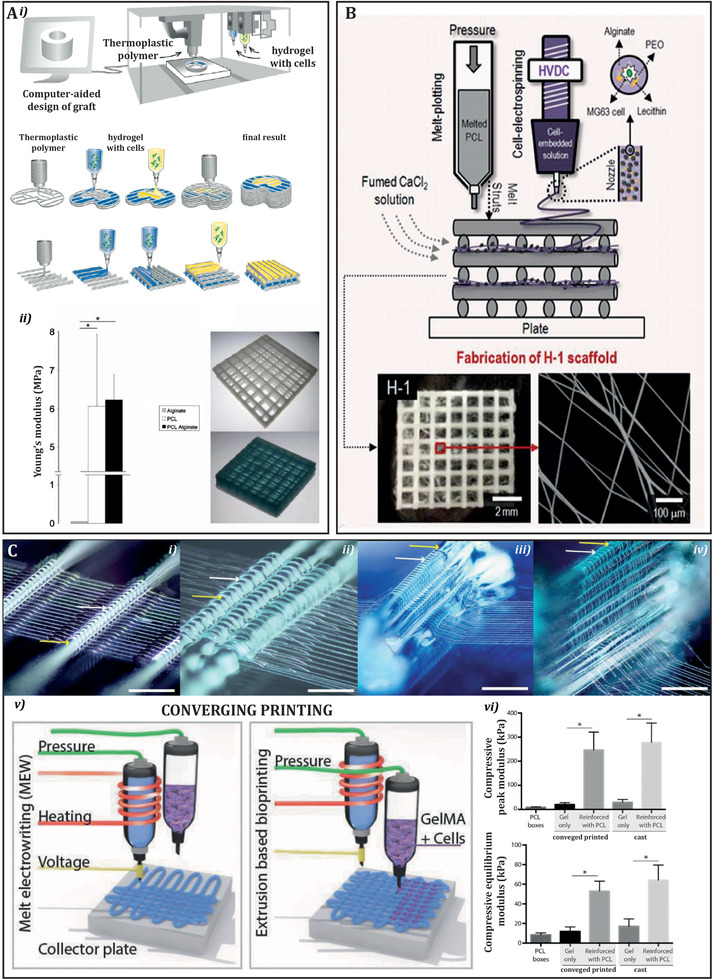
A) i) Schematic overview of the hybrid bioprinting process. ii) Mechanical properties of printed constructs. Young's modulus is shown for constructs composed of alginate, PCL and PCL combined with alginate. Examples of tested samples are shown: an empty PCL scaffold (top) and a PCL scaffold filled with alginate (bottom). Reproduced with permission.^[^ ^132^ ^]^ Copyright 2009, IOP Publishing, Ltd. B) Top: Schematic representation of converged fabrication process, consisting in microsize PCL structures using a melt‐plotting process and electrospun cell‐laden fibers. Bottom: optical and SEM images of the fabricated hybrid scaffolds. Reproduced with permission.^[^ ^144^ ^]^ Copyright 2015, Elsevier. C) Fiber reinforced hydrogels using hydrogel (Pluronic 40% w/v) to guide the direction of MEW (PCL) fibers; i) MEW fibers are guided over a single stand of hydrogel; ii) interlocked with hydrogel; iii) This enables more complex fiber architectures; and iv) out‐of‐plane fiber deposition. Yellow arrows depict the hydrogel whereas the white arrows depict the PCL fiber (scale bar = 500 mm). v) Schematic of MEW and extrusion‐based bioprinting process converged in a single‐step approach; vi) Mechanical properties of converged printed scaffolds compared with cast counterpart. No differences were found between the two. Reproduced with permission.^[^ ^30^ ^]^ Copyright 2018, Wiley‐VCH GmbH.

Up until now, combination of reinforcing thermoplastic materials with soft cell laden hydrogels required a two‐step cast approach.^[^ ^125^, ^126^, ^127^, ^128^, ^129^, ^130^, ^131^ ^]^ This considerably limits simultaneous control over reinforcing and cell laden hydrogel materials. Therefore, it has become apparent that the combination of different biofabrication techniques may be required to capture the complex composition and functionality of biofabricated tissues.^[^ ^123^, ^124^ ^]^ One of the first works on this direction was reported Schuurman et al. and team. The authors integrated this concept of biofabrication technologies hybridization by combining the layer‐by‐layer deposition of the rigid polymer (PCL) and the extrusion‐bioprinting of a cell‐laden hydrogel (alginate) (**Figure** **7**A). Interestingly, they demonstrated that the physical (thermal) requirements of the thermoplastic could be made compatible with the requirements needed to maintain cell viability in the hydrogel.^[^ ^132^ ^]^ This technique not only allowed to generate printed structures based on different biomaterial ink, but also facilitated the spatial inclusion of different cell‐types. The combination of alginate with PCL showed a significantly higher Young's modulus, which could be tailored by changing spacing, orientation, and/or thickness of the reinforcing fibers.^[^ ^132^ ^]^ This approach was also implemented by Stichler et al. who investigated the use of allyl‐functionalized poly(glycidol)s (P(AGE*‐co‐*G)) as a cytocompatible crosslinker for thiol‐functionalized hyaluronic acid (HA‐SH), which was co‐printed with a thermoplastic material (PCL) to ensure the overall mechanical integrity of the constructs.^[^ ^133^ ^]^ The mechanical stiffness of hydrogel component could be tuned by varying several parameters, including the hydrogel‐precursor concentration or the supplementation of the bioink with non‐crosslinking polymeric additives.^[^ ^133^ ^]^ The fabrication of reinforced cell laden hydrogel constructs by the combination of printing strategies has been particularly investigated for cartilage repair approaches, as these implants need to withstand high compressive and shear forces generated within the joints.^[^ ^134^ ^]^ For example, Mouser et al. explored the co‐printing of methacrylated poly[N‐(2 hydroxypropyl) methacrylamide mono/dilactate] polyethylene glycol (pHPMA‐lac‐PEG) and methacrylated hyaluronic acid hydrogel with PCL, demonstrating mechanical rigidity of the structures.^[^ ^135^ ^]^ Although the study highlighted the importance of proper integration of the PCL skeleton, interactions at the hard‐soft material interface were not investigated.^[^ ^135^, ^136^ ^]^ Moreover, the simultaneous extrusion‐based printing of a stabilizing network of a thermoplastic polymer and a cell‐laden bioink was also presented for the fabrication of reinforced tubular structures for reconstruction of the urethra using urothelial cells (UCs) and smooth muscle cells (SMCs).^[^ ^145^ ^]^ Kang et al. extended this approach to simultaneously print various cell‐laden composite bioinks (i.e., based on gelatine, fibrinogen, hyaluronic acid, and glycerol) and a synthetic biodegradable polymer (PCL) for the generation of human scale tissue constructs with potential personalized shape.^[^ ^146^ ^]^ This work demonstrated that complex 3D structures can be produced with multiple cell types and biomaterials, with the potential to form various types of vascularized tissue in vivo.^[^ ^146^ ^]^

Furthermore, a more recent development is the convergence of MEW with extrusion based bioprinting in a single printing platform.^[^ ^137^, ^138^ ^]^ Some of the important features that distinguish MEW from other filament reinforcing techniques are the improved resolution and the ability to integrate multimodal diameters in a single fabrication step.^[^ ^139^ ^]^ This technique allows to obtain stable structures with a porosity higher than 80% that encompass fibers with diameters between ≈300 nm and 50 µm,^[^ ^140^ ^]^ which approximate to the sizes of the fibrous structure of native ECM structure, and can subsequently significantly contribute to the mechanical properties of composite matrices.^[^ ^139^, ^141^, ^142^ ^]^ Important to mention that a first step in convergence of fiber formation fabrication technologies with biofabrication was reported by Kim et al. which integrating solution electrospinning of SF with melt electrospinning of PCL,^[^ ^143^ ^]^ while Yeo et al. proposed an alternative hybrid approach that combined melt spinning of PCL with electrospinning of cell‐laden alginate‐based bioink (Figure 7B).^[^ ^144^ ^]^ Nevertheless, extrusion‐based bioprinting (using GelMA as bioink) has been successfully converged with MEW of thermoplastic polymers (e.g., PCL) to create biologically functional constructs with more complex architecture and low thermoplastic content than the ones obtained by conventional extrusion printing. Importantly, the mechanical properties of the composite constructs did not differ from cast samples, meaning that reinforcing effect is not affected by the converged printing approach (Figure 7C).^[^ ^30^ ^]^

In the field of osteochondral regeneration, Diloksumpan et al. have used a similar converged bioprinting approach, in order to manufacture mechanically stable ostechondral implants that allow proper integration between cartilage and bone.^[^ ^147^ ^]^ This integration was particularly challenging as the cartilage phase was based on a soft reinforced hydrogel^[^ ^107^ ^]^ and the bone phase was based on a more brittle calcium phosphate (CaP) biomaterial ink.^[^ ^147^ ^]^ Micro‐fibrous polymeric networks, obtained via MEW, were incorporated into a methacrylate gelatin‐based hydrogel loaded with chondrogenic cells, and then anchored to the ceramic ink. While tested in vivo in a horse, results demonstrated successful mechanical stability and integration with surrounding native cartilage and bone tissues.^[^ ^148^ ^]^ This interesting converged bioprinting and biomaterials approach shows promise for engineering mechanical competent soft‐hard tissue interfaces.^[^ ^147^ ^]^

Thus, we have exemplified how the combination of fiber or filament reinforcing technologies with extrusion based biofabrication techniques has proven to be an useful fabrication approach for the realization of 3D constructs that can approximate to the structural and mechanical functionality of native tissues. However, optimizing the interfacial interactions between the reinforcing materials (fibers or filaments) and cell‐laden hydrogels still remains an open challenge, that when solved, could lead to the development of composite constructs with superior properties.

## 5 Conclusions and Future Prospectives

Biofabrication has been rapidly evolving in the last decade and, by developing methods for spatially coding 3D structures based on materials suitable for cell culture, it has gained great interest in the field of regenerative medicine.^[^ ^1^, ^6^, ^149^, ^150^ ^]^ Driven by the developments in additive manufacturing techniques, it offers the possibility to control not only the external geometry of a 3D construct, but also the internal microarchitecture, allowing precise control over the properties of scaffolds.^[^ ^151^ ^]^ In general, all biofabrication techniques aim to create 3D structures that mimic cellular physiological micro‐ and macro‐environments; however most strategies produce small‐scale scaffolds that enable cell proliferation for relatively short periods of time.^[^ ^28^ ^]^ Moreover, no technique alone manages to simultaneously recreate the adequate microenvironment and mechanical properties typical of a native tissue yet.^[^ ^20^ ^]^ Therefore, the next generation of biofabrication techniques will have to monitor multiple aspects, and as a result research has currently been focused on creating new biomaterials and combination of different biomaterials and 3D printing processing technologies that can better replicate the complex and heterogeneous nature of native tissues and organs.^[^ ^149^ ^]^

In this review, we have summarized current strategies to mechanically reinforce soft biomaterials, while paying particular attention to the interactions at the interface between reinforcing materials and soft hydrogels. These interactions play an important role in the final mechanical and biological properties of a multimaterial, composite scaffold. Ideally, the mechanical, physical, and biological properties of biomaterials should be considered altogether when designing the microarchitecture of biofabricated structures. This, however, is not so straightforward due to the low number of available materials compatible with these manufacturing techniques and biomedical applications.

**Figure 8:**
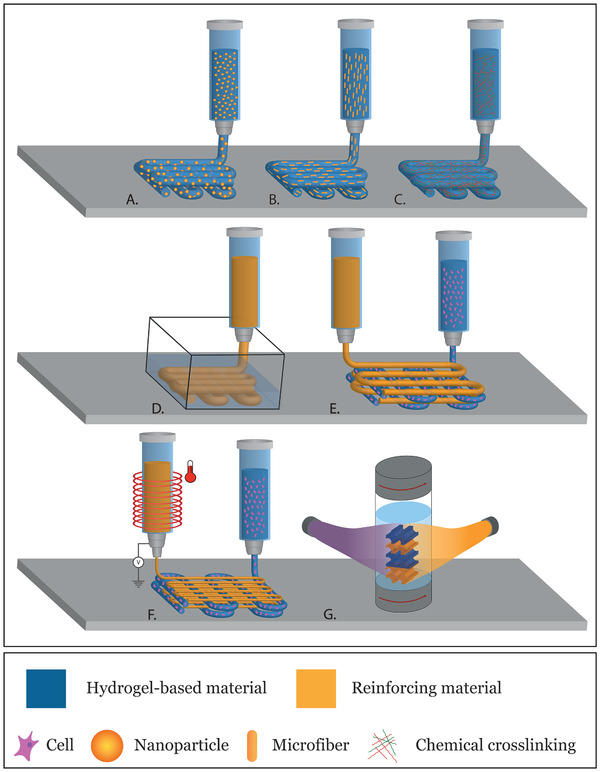
Summary of existent and envisioned 3D (bio) printing techniques of composite materials. Extrusion printing of hydrogel‐based material with A) nanofillers, B) microfibers, or of C) IPN or DN based hydrogels. Extrusion printing D) inside a support bath or E) with other extruded printing support materials. F) Combination of extrusion printing with fiber reinforcing materials, for example obtained by meltelectrowriting, within in a single bioprinting platform. G) Envisioned perspective on combining reinforcing materials with light assisted, volumetric 3D bioprinting techniques, of hydrogel based materials and reinforcing structures.

From the literature review presented here, we observed that the use of nanofillers, as well as microfibers, to reinforce hydrogel‐based matrices (**Figure** **8**A,B) confers an enhanced mechanical performance compared to conventional hydrogels alone, especially when it is possible to exploit the synergistic interaction between the matrix and the reinforcement. However, numerous studies have been carried out to understand the in vitro and in vivo behavior of these nano‐ and micro‐composites and, although there are several methods of synthesis, cyto‐ and biocompatibility are not always guaranteed. Many factors still need to be improved, including the interaction at the interface with the hydrogel‐based matrix. On the other side, the use of IPNs and DNs is an interesting method to recreate dynamic networks, such as the ones found in the ECM structure of native living tissues (Figure 8C). These intertwined networks have enormous potential and can be further developed to recreate not only structures sufficiently robust to withstand mechanical stress, but also structures that incorporate bioactive molecules and promote tissue growth or repair. Furthermore, we have reviewed how the cell‐laden hydrogels have been combined with reinforcing structures. What emerges from our literature research, is that 3D printing allows to generate reinforced constructs with an increasing degree of geometrical complexity and improved mechanical performance (Figure 8D). However, encapsulation of viable cells is not trivial, and consequently extrusion bioprinting has been introduced as a combined technology (Figure 8E). In addition, the developments on fiber printing technologies like MEW, represented a step forward with respect to the incorporation of 3D reinforcement structures within cell‐laden hydrogels that did not compromise neo‐tissue deposition (Figure 8F). These innovative reinforcing strategies are becoming more frequent in tissue engineering but, with some notable exceptions, have not adequately addressed the challenges of combining reinforcing structures and hydrogels. Last, opportunities related to the convergence of other 3D (bio)printing technologies, like volumetric bioprinting of different material compositions (Figure 8G), or eventually with other complementary fabrication technologies, may address specific challenges for composite materials fabrication, but will raise additional challenges on engineering materials interfaces.

Importantly, we observed that the introduction of an engineered interface, both through induction of physical interactions and through chemical modifications, has proved to be an excellent method for solving the problem of poor compatibility between reinforcing components and hydrogel‐based matrices. These interactions tend to reduce the interface energy and improving the mechanical properties. While most of the studies reviewed reported some improvements on the interface bonds strength, important challenges still remain open. First, some components used for interface modification (such as catalysts or surfactants) can cause toxicity to cells and tissues if not removed properly. Here, atmospheric pressure plasma technologies can be used as an alternative strategy to dramatically improve interactions with cells, to improve compatibility between reinforcing polymer structures surfaces and hydrogels, and to activate them for covalent immobilization of biomolecules. Second, the formation and breaking mechanism of such bonds must be further investigated to evaluate how far the improvement of the structural, mechanical, and biological properties of the scaffold can go. Third, reliable ex vivo studies, which approximate in vivo conditions, are greatly needed to evaluate future clinical translation of composite materials with engineered interfaces. In conclusion, taking interface interactions into account when creating composite materials and bioprinted constructs is essential for the creation of functional, both in terms of mechanical and biological properties, living materials for regenerative medicine applications.

## Conflict of Interest

The authors declare no conflict of interest.
